# A Perspective Around Cephalopods and Their Parasites, and Suggestions on How to Increase Knowledge in the Field

**DOI:** 10.3389/fphys.2018.01573

**Published:** 2018-11-20

**Authors:** Katina Roumbedakis, Marie Drábková, Tomáš Tyml, Carlo di Cristo

**Affiliations:** ^1^Dipartimento di Scienze e Tecnologie, Università degli Studi del Sannio, Benevento, Italy; ^2^Association for Cephalopod Research, Naples, Italy; ^3^Department of Parasitology, Faculty of Science, University of South Bohemia, České Budějovice, Czechia; ^4^Institute of Parasitology, Biology Centre Academy of Sciences of the Czech Republic, České Budějovice, Czechia; ^5^Department of Botany and Zoology, Faculty of Science, Masaryk University, Brno, Czechia

**Keywords:** Cephalopoda, parasites, pathogens, diseases, welfare

## Abstract

Although interest in several areas of cephalopod research has emerged over the last decades (e.g., neurobiology, aquaculture, genetics, and welfare), especially following their 2010 inclusion in the EU Directive on the use of animals for experimental purposes, knowledge regarding the parasites of cephalopods is lacking. Cephalopods can be intermediate, paratenic, or definitive hosts to a range of parasites with a wide variety of life cycle strategies. Here, we briefly review the current knowledge in cephalopod parasitological research, summarizing the main parasite groups that affect these animals. We also emphasize some topics that, in our view, should be addressed in future research, including: (i) better understanding of life cycles and transmission pathways of common cephalopod parasites; (ii) improve knowledge of all phases of the life cycle (i.e., paralarvae, juveniles, adults and senescent animals) and on species from polar deep sea regions; (iii) exploration of the potential of using cephalopod-parasite specificity to assess population boundaries of both, hosts and parasites; (iv) risk evaluation of the potential of standard aquacultural practices to result in parasite outbreaks; (v) evaluation and description of the physiological and behavioral effects of parasites on their cephalopod hosts; (vi) standardization of the methods for accurate parasite sampling and identification; (vii) implementation of the latest molecular methods to facilitate and enable research in above mentioned areas; (viii) sharing of information and samples among researchers and aquaculturists. In our view, addressing these topics would allow us to better understand complex host-parasite interactions, yield insights into cephalopod life history, and help improve the rearing and welfare of these animals in captivity.

## Cephalopods and Their Parasites: A Short Overview

The incidence of a given parasite in a cephalopod species depends on the presence of a potential definitive host and intermediate host(s) (in parasites with complex life cycles, i.e., those that use multiple hosts to complete their life cycle), as well as on biotic and abiotic factors (González et al., 2003). Cephalopods can be definitive hosts for protists, dicyemids, monogeneans and crustaceans, as well as intermediate or paratenic hosts for digeneans, cestodes and nematodes (summarized in Table 1; for review see also Table 1–5, Hochberg, 1990). As intermediate or paratenic hosts, cephalopods can accumulate parasites throughout their lifespan, thus increasing the chance of predation by the next host and, consequently, the probability of parasite transmission. This is especially relevant for cestodes and anisakid nematodes, which use cephalopod hosts as important vectors for transporting them to other intermediate or to definitive hosts (e.g., Pascual et al., 1995; Abollo et al., 1998; Petrić et al., 2011).

**Table 1:** Parasitic taxa (approximately 230 parasites identified at species level) infecting cephalopods (sorted by order) reported in the literature to date.

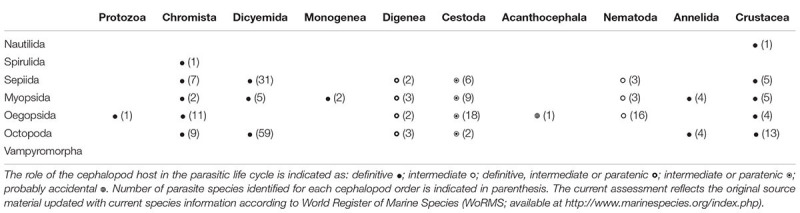

In contrast to other molluscs, two characteristics of coleoid cephalopods (all living cephalopods besides *Nautilus* spp.) have crucial roles in their susceptibility to parasites and disease: (i) the loss of external shell, which enables the extensive neural and muscular development that allows high-speed locomotion; and (ii) the evolution of complex skin capable of sophisticated camouflage and signaling, but also prone to lesioning (Kinne, 1990). By shedding the rigid external shell of their ancestors, coleoids became more agile predators and adopted a more active lifestyle. This likely increased the frequency of parasite transmission since, predators readily accumulate multi-host parasites that are transmitted upward through the food web (e.g., digeneans, cestodes and nematodes). Some parasites can even alter the behavior or appearance of their intermediate hosts (e.g., modifying host phenotypes) in order to increase the likelihood that they will be predated on by their definitive hosts (Lafferty, 1999; Heil, 2016), mechanisms that have yet to be explored in cephalopod hosts. In addition to the increased likelihood of transmission, the fragility of coleoid cephalopods’ skin may increase the ease with which opportunistic pathogens (i.e., infection by bacteria, kinetoplastids, dinoflagellates, fungi, labyrinthulids) can invade the body (reviewed by Kinne, 1990).

To date, the most complete review of potential pathogenic agents affecting cephalopods is in “Diseases of Marine Animals” (DoMA; Kinne, 1990; chapters concerning cephalopods: Hanlon and Forsythe, 1990a,b; Hochberg, 1990). In his summary, Hochberg (1990) reported parasites for about 130 cephalopods, which represents less than a quarter of the described species at that time. Later reviews provided complementary information regarding the main viral, bacterial, fungal, parasitic, chemical and mechanical parasitic agents affecting cephalopods (see Pascual et al., 1996; Castellanos-Martínez and Gestal, 2013; Sykes and Gestal, 2014).

**FIGURE 1:**
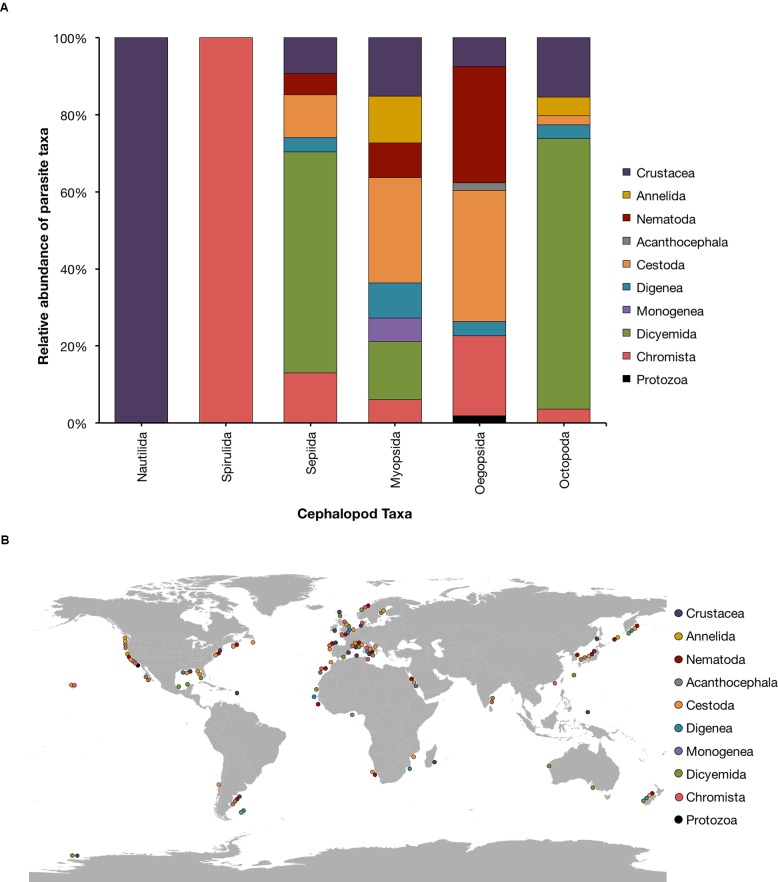
**(A)** Relative abundance of parasitic taxa affecting cephalopods. **(B)** Place of capture of the cephalopod hosts. The current assessment reflects the original source material updated with current species information according to World Register of Marine Species (WoRMS; available at http://www.marinespecies.org/index.php).

In the following paragraphs, we briefly overview the current knowledge on the most common parasites found in cephalopods. About 230 parasitic species of a variety of taxa (e.g., Chromista, Protozoa, Diciemyda, Monogenea, Trematoda, Cestoda, Acanthocephala, Nematoda, Annelida and Crustacea) are reported in the literature to date (Table 1 and Figure 1A). A map of the geographic distributions of cephalopod parasites is provided in Figure 1B. We emphasize that the data provided here likely over-represents tropical and temperate locations and coastal environments, since these areas are more easily and frequently sampled.

### *Aggregata* spp.

Some of the most common parasites of cephalopods are the coccidians *Aggregata* spp. (Apicomplexa, Aggregatidae). To date, 10 species of *Aggregata* have been described parasitizing cephalopods (for review, see Gestal et al., 2010), although other (undescribed) species have also been reported (reviewed in Hochberg, 1990), so the actual diversity is likely higher. *Aggregata* spp. have complex heteroxenous life cycles, with crustaceans as intermediate hosts and cephalopods as definitive ones (Dobell, 1925; Hochberg, 1990). Most recent research (e.g., Castellanos-Martínez et al., 2013; Tedesco et al., 2017) has focused primarily on *Aggregata octopiana* and *Aggregata eberthi*, parasites of *Octopus vulgaris* and *Sepia officinalis*, respectively. This group is associated with histological and ultrastructural lesions in the digestive tract (mainly the caecum and intestine) of their cephalopod hosts (Gestal et al., 2002a), with infections of the gills, mantle, arms and mesentery also occasionally occurring (Pascual et al., 1996; Mladineo and Bočina, 2007; Tedesco et al., 2017). In addition, *Aggregata* infection can impair body growth due to “malabsorption syndrome” (Gestal et al., 2002b).

### Ciliates and Dicyemids

In the renal tissue, cephalopods harbor two very unique parasitic groups, the apostome ciliates, *Chromidina* spp., and metazoans Dicyemida ( = Rhombozoa). Five *Chromidina* spp. and over one hundred dicyemids have been described infecting cephalopods (Catalano, 2012; Souidenne et al., 2016). The exact impact on the hosts is still uncertain; for instance, in *O. vulgaris*, low levels of tissue abrasion caused by dicyemids could be observed by electron microscopy (Ridley, 1968), but no impact was detectable using light microscopy (Furuya et al., 2004). Consequently, these organisms may eventually come to be considered symbiotic rather than parasitic (Katayama et al., 1995; Furuya et al., 2004). Bacterial symbionts are also observed in cephalopods: for instance, the bacteria colonizing the pericardial appendage of *Nautilus* sp. (Pernice et al., 2007; Pernice and Boucher-Rodoni, 2012) as well as the well-established association between *Euprymna scolopes* and *Vibrio fischeri* (Ruby, 1999, for review see Gerdol et al., 2018). Further studies of such symbiosis can improve not only our understanding of these complexes associations in cephalopods, but also give insights on how bacterial symbiosis occurs in mammals (Gerdol et al., 2018).

### Monogeneans

A few studies have reported monogenean parasites in cephalopods (see Sproston, 1946; Palombi, 1949; Dollfus, 1958; Bychowsky, 1961). The gyrodactylid *Isancistrum subulatae* has been found in the arms and tentacles while *Isancistrum loliginis* in the mantle cavity and gills of *Alloteuthis subulata* (Llewellyn, 1984). Identifying monogeneans in cephalopods is extremely difficult due to their delicateness, small size and the thick layer of mucus in cephalopod tissues (Llewellyn, 1984), and this could be the reason for their supposed rarity. In the future, potential sites of occurrence (e.g., arms/tentacles, mantle, funnel and gills) should be thoroughly examined for a better assessment of their true prevalence.

### Digeneans

The majority of information regarding digenean parasites of cephalopods is provided by Overstreet and Hochberg (1975) and Hochberg (1990), with some information added over the following decades (e.g., Shukhgalter and Nigmatullin, 2001; Nigmatullin et al., 2009), including digenean records in squid paralarvae (Vidal and Haimovici, 1999). Around 20 species have been reported from nearly 30 cephalopod hosts, usually with low prevalence of infection (Hochberg, 1990). Cephalopods do not seem to play a major role in digenean life cycles (Hochberg, 1990), though our knowledge is too limited to support this premise definitively.

### Cestodes

Cephalopods are second and/or third intermediate or paratenic hosts for cestodes, acting as important vectors transporting them to other intermediate (e.g., cetaceans; Aznar et al., 2007) or definitive hosts (e.g., elasmobranchs and fishes; Hochberg, 1990). Several species have been reported in around 60 cephalopod hosts: larval and post-larval cestodes from the orders Trypanorhyncha and Tetraphyllidea are commonly found freely in cephalopod digestive tracts, usually the stomach, caecum and intestine (Hochberg, 1990). However, they can also be found in the buccal mass (in octopus; Roumbedakis, unpublished data) or encysted in the digestive tract, mesentery and mantle cavity (Hochberg, 1990). *Phyllobotrium* spp. is the most frequently reported species (Hochberg, 1990). A general life cycle for Phyllobothriidae has recently been suggested (Klotz et al., 2018): procercoid development occurs in crustaceans (first intermediate hosts), followed by plerocercoid development in bony fish, sea turtle or squid (second intermediate host). Marine mammals can harbor both plerocercoids and merocercoids, acting as third intermediate or paratenic hosts, and sharks serve as the definitive hosts, harboring the adult parasites.

### Nematodes

Larval nematodes are commonly found encysted in the viscera and musculature of cephalopods (Hochberg, 1990; Gestal et al., 1999; Abollo et al., 2001), making infected animals aesthetically unattractive for human consumption (Smith and Wootten, 1984). *Anisakis* (Anisakidae) is one of the most abundant and frequent cephalopod parasites causing important pathological effects to their hosts, such as ulceration (Abollo et al., 2001), and even castration if encysted in the gonads (Abollo et al., 1998). Transmitted through food webs, these parasites have complex life cycles involving multiple hosts: planktonic or bentho-planktonic crustaceans are the first intermediate hosts; fish and squids act as second intermediate or paratenic hosts and marine mammals (mainly cetaceans) as definitive hosts (Mattiucci and D’Amelio, 2014; Mattiucci et al., 2018). To date, a number of cephalopods (*S. officinalis*, *Ancistroteuthis lichtensteinii*, *Histioteuthis bonnellii*, *Illex coindetii*, *Todarodes sagittatus*, *T. pacificus*, *Todaropsis angolenis*, *T. eblanae*, *Nototodarus sloanii*, *Dosidicus gigas*, and *Moroteuthis ingens*) are known to be parasitized by six of the nine *Anisakis* species (*A. simplex*, *A. berlandi*, *A. nascettii*, *A. pegreffii*, *A. physeteris*, and *A. typica*) currently described (for review see Tables 2–5, Mattiucci et al., 2018). Recent advances in anisakid biology and systematics are comprehensively summarized by Mattiucci et al. (2018). It is also worth noting that humans may also become accidental hosts if live larvae of *Anisakis* spp. are ingested through the consumption of raw or undercooked infected squid and cuttlefish. Additionally, even when ingested dead, *Anisakis* larvae can induce allergic reactions (Audicana et al., 2002; Mattiucci et al., 2013) or gastrointestinal problems (Audicana et al., 2002). Although rare, anisakiasis (the infection of a human by this parasite) is likely underdiagnosed and thus underestimated worldwide and may pose a greater threat to public health in the future (Bao et al., 2017; Mattiucci et al., 2018).

### Crustaceans

Crustaceans, primarily copepods and isopods, usually parasitize the gills and mantle cavities of coleoid cephalopods (Pascual et al., 1996), but can also parasitize external surfaces, such as arms or head (Hochberg, 1990). Some attention was lately focused on tisbid copepods, parasites of deep-sea octopods. The details of the *Cholidya polypi* morphology and life cycle as well as a summary of Tisbidae infecting octopods are provided by Humes and Voight (1997), while a genus/species with an endoparasitic life stage infecting *Vulcanoctopus hydrothermalis* is described by López-González et al. (2000).

## Cephalopod Parasitology: Suggestions for the Future

Despite an increase in the understanding of cephalopod parasitology during the last decades, there are still many gaps in current knowledge. Here, we briefly discuss what we believe to be the most critical issues/questions for basic and applied research that require attention.

### Parasite Life Cycles and Transmission Pathways

The life cycles and transmission pathways of many cephalopod parasites are still unclear. For instance, the methods of dicyemid transmission are completely unknown (Catalano et al., 2013), and it has been estimated that less than 5% of the life cycle of marine helminths has been fully described (Poulin et al., 2016). In the case of helminths, accurate identification of these parasites by classical methods depends on the features of adult parasites, which normally occur in vertebrates. However, the adult stages of larval helminths are frequently unknown (Aznar et al., 2007), partially due to disparity in the number of parasitological studies of invertebrates compared to vertebrates (Poulin et al., 2016). Molecular tools combined with phylogenetics can help identify trophic interactions that lead to the transmission of parasites and to a better understanding of parasite life cycles (e.g., Randhawa and Brickle, 2011). Also, our understanding of interactions between diet, feeding behavior, parasitic disease, and transmission pathways of cephalopod parasites can be improved with similar combinations of traditional approaches and modern molecular methods (e.g., Petrić et al., 2011).

### Poorly Explored Life-Stages and Species From Polar and Deep Sea Regions

Most of the cephalopod parasites have been described in shallow-water species. Emerging exploration of polar and deep-sea will likely expand our knowledge about the diversity of cephalopod parasites. Similarly, the current knowledge is largely restricted to juvenile and adult cephalopod hosts, with few parasites known for paralarvae/early juveniles (Vecchione, 1987; Vidal and Haimovici, 1999) and senescent animals (Pascual et al., 2010). The extension of these limits (geographical-, life-stage-, and habitat-wise-) may be the basis for new insights into host-parasite relationships, offering important insights about the parasite diversity and complexity.

### Cephalopod Parasites as Biological Tags in Population Studies

Studies of parasite distribution and host specificity can provide information about host population structure, phylogeographic distribution, migration patterns and general biology. Insights into host specificity can also help predict the likelihood of a parasite successfully establishing itself and spreading in new populations, geographical regions and hosts (Poulin and Mouillot, 2003), a possibility which becomes increasingly important with accelerating global climate change.

Parasites are often utilized as “tags” for fisheries stock assessment, especially in small populations and limited timescales (MacKenzie, 1999; Mattiucci et al., 2015). *Anisakis* have been used as biological markers to identify sub-populations of pelagic and demersal fishes from the Mediterranean Sea (for review, see Mattiucci et al., 2015). In cephalopods, such studies are rare, mainly targeting squids (reviewed in Pascual and Hochberg, 1996; Catalano et al., 2014b). Although taxonomy within this clade is not yet well resolved (see Catalano, 2012 for review), dicyemids could serve the same purpose for certain benthic cephalopods, since they are closely bound to their hosts and differ across the hosts’ geographical range (Catalano et al., 2014a). Another promising taxon is *Aggregata*, which, in the Mediterranean, is differentiated into three distinct clades, potentially reflecting population differentiation of its widespread host, *O. vulgaris* (Tedesco et al., 2017).

### Possible Parasite Outbreaks in Cephalopod Aquaculture

Cephalopod parasites rarely cause mortality or serious damage to wild populations. However, synergic effects between different stressors associated with captivity may favor parasites and other pathogens, making parasite outbreaks more likely in aquaculture. Coincident with the development and proliferation of aquaculture, parasites and other pathogens have proliferated (e.g., Overstreet, 1973; Lom and Dyková, 1992), many causing serious economic and environmental problems. Although our knowledge of cephalopod parasites in captivity is limited, we can extrapolate (with some caution) from knowledge obtained from other, already well-established, marine organism cultures.

In fish culture for instance, high population density is known to favor rapid spread of infections, especially those caused by parasites with direct life cycles, such as monogeneans and caligid copepods (e.g., Thoney and Hargis, 1991; Johnson et al., 2004). Both groups have already been reported in cephalopods (e.g., Llewellyn, 1984; Pascual et al., 1996), and are thus worth monitoring particularly attentively in cephalopod aquaculture. High-density culture of hosts can also disrupt an otherwise stable parasite life-cycle scheme. For example, the myxosporeans *Enteromyxum* spp. normally alternate between two hosts (fish and annelid), but are known to be capable of direct fish-to-fish transmission in high-density conditions (Diamant, 1997). Likewise, another group of myxosporeans, *Kudoa* spp., which have been reported in wild octopus populations and are known to cause serious problems for marine fish aquaculture (Moran et al., 1999), has been suggested as a potential parasite in cephalopod culture (Yokoyama and Masuda, 2001). *Aggregata octopiana*, despite having a complex life cycle, can also impact octopus health during commercial ongrowing (Gestal et al., 2007).

In captivity, even apparently harmless symbionts, such as dicyemids and *Chromidina* spp., can become pathogens and inflict tissue damage to debilitated cephalopods (e.g., blocking the renal sacs ducts, Sykes and Gestal, 2014). At least three phylogenetically distant groups of potential eukaryotic pathogens that are capable of both a free-living and parasitic lifestyle (termed also saprophagic) can also be considered as potential pathogens of cephalopods: histophagous ciliates, known from cultured fish, crustaceans and bivalves (e.g., Cawthorn et al., 1996); amphizoic amoebae, known from cultured fish, crustaceans, bivalves and sea urchins (e.g., Dyková and Lom, 2004); and various fungal-like organisms known from cultured fish, crustaceans and molluscs (e.g., Derevnina et al., 2016). Since these pathogens are not limited by trade-offs regarding transmission or virulence because of their independent free-living stage (Kuris et al., 2014), they usually cause devastating economic impacts in aquaculture. Several ‘fungus-like organisms’ and histophagous ciliates have already been reported from cephalopods (Hanlon and Forsythe, 1990a; Tao et al., 2016) but, to date, no amphizoic amoebae have been identified.

### Standardization of Parasite Sampling and Identification

Standardization of the sampling and identification methods used for cephalopods is required. Given the particular anatomy of the different cephalopod species, the publication of a guidelines, that could be used for example for parasitological and health status assessment of kept cephalopods or to determine their cause of death, would greatly facilitate research. For parasite identification, the use of classical methods (e.g., using taxonomic keys) can be extremely difficult for larval stages (Catalano et al., 2014b) or for species with high level of morphological plasticity (Poulin and Morand, 2000). In addition, some of the original parasite descriptions are not available in English (e.g., dicyemids, Nouvel, 1947, 1948; Van Beneden, 1876; Bogolepova-Dobrokhotova, 1953, 1960, 1962), are sometimes, incomplete (see Furuya, 2007), and often muddled by a variety of unresolved taxonomic and nomenclatural issues (e.g., nematodes, Smith and Wootten, 1978) which impair precise parasite identification.

The use of alternative approaches, such as search for additional morphological characters that complement classical parasite identification as suggested by Tedesco et al. (2017), the use of genetic and molecular techniques (e.g., Kopečná et al., 2006; Castellanos-Martínez et al., 2013; Souidenne et al., 2016; Tedesco et al., 2017), as well as combinations of multiple methods, is growing. Such approaches should help to better elucidate and re-evaluate the taxonomic status and host-parasite relationships, particularly where morphological plasticity might be of concern (Pascual et al., 2007). Moreover, it may clarify relationships within species complexes, such as that of *A. octopiana* infecting *O. vulgaris* in Mediterranean areas (Tedesco et al., 2017). Finally, taxonomic review of genera with morphological descriptions and molecular markers would aid research and improve assessment methods for cephalopod health and food safety in aquaculture.

The use of non- or minimally invasive methods for *in vivo* detection of cephalopod parasites should be explored in the near future. For instance, it has been suggested that *Aggregata* infection could be diagnosed through the presence of sporocysts in the feces of living animals or through inspection of the terminal intestine by gentle retraction of the ventral mantle or by endoscopy (Sykes et al., 2017). Detection of cephalopod parasite infection using ultrasound imaging or swabbing for parasite molecular/DNA sampling might also be possible. The development of these methods would facilitate early diagnosis, ultimately preventing disease outbreaks and improving animal welfare in captivity.

### Cutting Edge Molecular Methods

Transcriptomics, genomics and proteomics (“omics”) are relatively new tools for understanding direct host parasite relationship on a molecular level. By enabling the study of the microbiome and metagenome of different cephalopod organs in relation to parasitic infection, the consequent pathology and immune response of hosts can be better understood (see for example Castellanos-Martínez et al., 2014a,b). Additionally, low coverage genome re-sequencing or reduced representation sequencing (RADseq methods, Davey and Blaxter, 2010) provide a tool for probing the genomic structure of populations with an unprecedented level of clarity for both host and parasites. Ultimately, such genomic information coupled with environmental data results in a “seascape genomics” approach, which can reveal both local genetic adaptations as well as the broader dynamics of gene flow (Riginos et al., 2016).

### Effect of Parasites in Cephalopod Physiology and Health

Host responses to parasites may involve a variety of physiological mechanisms (e.g., neural, endocrine, neuromodulatory and immune) that can interact and alter host behavior (see review in Thompson and Kavaliers, 1994). For example, in fishes, parasitism can cause conspicuous host behavior (e.g., impaired sensory and swimming performance, increased time at water surface, etc.), increasing predation risk (Lafferty and Morris, 1996). Parasites can also affect fish performance in terms of growth and reproduction, consequently impacting their health and welfare (Barber, 2007). Unfortunately, in cephalopods, the effects of parasitism are usually reported only at histopathological level, whereas physiological and behavioral effects are virtually unexplored. Experimental studies combining both behavioral and quantitative physiological indicators will help to better understand host-parasite systems and, hopefully, enable better assessment of cephalopod welfare. New technologies such as “omics” approaches and electron and florescent microscopy will certainly facilitate this research.

### Resource Sharing

Although researchers have been able to build on previous research to some extent (e.g., through examination of collection of parasites and voucher specimens kept in museums, or gene mining in NCBI genetic database), there is much to be gained from employing a more open approach. The sharing of material through lab networks or open databases can reduce research effort and cost, maximize data use, and minimize the number of animals sampled. This is especially relevant for animals difficult to obtain, such as deep-sea cephalopods.

A database of cephalopod parasites and their cephalopod hosts available from the scientific literature, as already published for other species (e.g., Global Mammal Parasite Database, www.mammalparasites.org), possibly with extension of curated database of molecular barcodes, should be considered. In this regard, efforts are currently underway to publish a free online database of parasites and other pathogenic agents of cephalopods, the “Cephalopods’ Pathogenic Agents Database (CephPAD),” which will include information on the affected tissue, anatomical-pathological findings, clinical presentation and mortality. An Atlas of Cephalopod Pathogens and Diseases is also in progress as follow-up to the activities of the COST Action FA1301. These initiatives will greatly facilitate the assessment of pathogenic agents and might facilitate early diagnosis of cephalopod pathogenic agents when they occur.

## Author Contributions

All authors contributed to the manuscript and approved the final version.

## Conflict of Interest Statement

The authors declare that the research was conducted in the absence of any commercial or financial relationships that could be construed as a potential conflict of interest.
